# 

**Published:** 1910-12-03

**Authors:** 


Just Issued. Ninth Edition, Rewritten and Enlarged, 9s. net.

**PHARMACY, MATERIA MEDICA & THERAPEUTICS**

Being a Commentary on the B.P. and its Drugs, with their Preparations, and on all the new Non-official Remedies in use, with Chapters on the different processes used in Dispensing, Prescription-writing, Latin Glossary, &c. By **Sir W. WHITLA, M.D., LL.D.,** Professor, Materia Medica, Queen's University, Belfast, and Senior Physician, Royal Victoria Hospital.

By the same Author. New Work. 1,900 pages. Crown 8vo. 25s. net.

**PRACTICE OF MEDICINE.**

Containing in two Handy Volumes a complete and independent Article on the Etiology, Symptoms, and Diagnosis of every Medical Disease; arranged in Dictionary Form for Practitioners and Students.

London: BAILLIÈRE, TINDALL & COX, 8 Henrietta Street, W.C.

KEGAN PAUL, TRENCH, TRÜBNER & CO., LTD.

### Sermon in the Hospital

BY MRS. HAMILTON KING.

| | | |
|---|---|---|
| F'cap 8vo. | In leather . . . . . | 2/- net. |
| F'cap 8vo. | In cloth . . . . . | 1/- |
| Miniature Edition. | In velvet calf . | 1/6 net. |
| Miniature Edition. | In leather . . | 1/- net. |

### Within Hospital Walls

BY LADY LINDSAY.

| | | |
|---|---|---|
| Miniature Edition. | In velvet calf . | 1/6 net. |
| Miniature Edition. | In leather . . | 1/- net. |

¶ Readers of *THE HOSPITAL* are invited to write to the Publishers for their *Illustrated Catalogue of Autumn Books.*

DRYDEN HOUSE, GERRARD ST., LONDON, W.

### Obituary.

The death is announced from Paris of Dr. Trousseau, son of the great clinician and oculist to the Quinze-Vingts, who was killed outright in a motor accident on October 31. The deceased was a distinguished and busy practitioner, and well known for his contributions to medical periodicals.

The death is announced of Mr. George Carrick Steet, late medical officer-in-chief to the General Post Office, at the age of ninety-two. Mr. Steet took his M.R.C.S. and L.S.A. in 1840, and nine years later became an F.R.C.S., having studied at St. George's Hospital. His appointments at different times included the post of surgeon to the Royal Maternity Charity, and to Queen Adelaide's Lying-in Hospital. He was also consulting medical officer to the Spanish Consulate in London. He was an occasional contributor to the medical papers.

Dr. G. de Gorrequer Griffith, of St. George's Square, S.W., has died at the age of seventy-three. He took his M.R.C.S. in 1860, and his L.R.C.P. and his L.M.Dublin in 1863. He was the founder and late senior physician of the Grosvenor Hospital for Women and Children. He was consulting physician to the Hounslow Hospital, physician to the Westminster Hospital for Women and Children, and formerly resident surgeon at the Home for the Diseases of Women and at the Lock Hospital. Since 1864 he had been editor of the "Hospital Mirror and Records" in the *Medical Press and Circular*. He was also a contributor to the medical journals.

The death took place on Monday, at Cliftonville, Belfast, of Surgeon-Major-General James Sinclair, K.H.P., late Army Medical Staff, who retired in 1892. He was educated at Edinburgh University, graduated M.D. in 1853 from the Royal College of Surgeons, and he obtained a commission in the Army Medical Service. In 1865 he was with the 33rd (Duke of Wellington's) Regiment at Bombay and Scinde, and again in Abyssinia, where he was present at the capture of Magdala in 1868. He was mentioned in despatches and gained the medal and promotion to the rank of surgeon-major. His appointments included those of principal medical officer Bermuda 1876-79, Belfast district 1880, South Africa and Transvaal 1881-82, Aldershot 1882, Ireland 1889, Malta 1884-88, Ireland 1889-92. He received also the Queen's Jubilee commemoration medal in 1897, and the Coronation medal in 1902. In 1896 he was appointed honorary physician to Queen Victoria.

Dr. John Ross Murray has died at Gipsy Hill, S.E. He had retired from the Army Medical Service, in which he had been brigade surgeon lieutenant-colonel. He studied at Edinburgh, where he graduated M.D., and became later F.R.C.S. of England.

The death is announced of Dr. David Dyce Brown, M.A., aged seventy. He was educated at the University of Aberdeen, where he graduated M.A. in 1859, and M.D. and C.M. in 1863. He had been assistant house surgeon at South Staffordshire General Hospital, medical officer at Aberdeen General Dispensary, and assistant Professor of Materia Medica at the University there. Later he was consulting physician to the London Homœopathic, Leicester Homœopathic, and Phillips Memorial Hospital, Bromley. His principal publication was "The Reign of Law in Medicine." For thirty years he acted as secretary to the annual Homœopathic Congress, and for the greater part of this time was co-editor of the *Monthly Homœopathic Review*. He established himself in homœopathic practice in London in 1876.

### Appointments and Resignations.

Mr. Joseph Cunning, M.B., F.R.C.S., has been appointed surgeon to the Cancer Hospital in place of Mr. C. H. Leaf, deceased. Mr. Cunning is assistant surgeon to the Royal Free Hospital and surgeon to the Victoria Hospital for Children.

Mr. C. Colgate Holman, M.B., B.C. (Cantab.), M.R.C.S., L.R.C.P., and Mr. M. M. Morrison, M.B., Ch.B. (Edin.) have been appointed assistant house surgeons to the Leicester Infirmary.

The following appointments have been made at the Manchester Royal Infirmary: Mr. R. A. Jackson, M.B., Ch.B. (Vict.), and Mr. G. M. Benton, M.B., Ch.B. (Vict.), to be senior house surgeons; Mr. A. E. Woodall, M.Sc., M.B., Ch.B. (Vict.), and Mr. T. T. Higgins, M.B., Ch.B. (Vict.), M.R.C.S., L.R.C.P., to be junior house surgeons; Mr. Alex. Porter, M.B., Ch.B. (Vict.), to be house surgeon to special departments; Mr. R. C. Hutchinson, M.B., Ch.B. (Vict.), Mr. James Cowan, M.R.C.S., L.R.C.P., and Mr. C. Y. Laing, M.R.C.S., L.R.C.P., to be house physicians; Mr. Ulick J. Bourke, L.R.C.P., L.R.C.S. (Edin.), to be assistant medical officer at Barnes Convalescent Home; and Mr. G. B. Warburton, M.B., Ch.B. (Vict.), to be accident room house surgeon.

### ANTI-TOXIN AND THE DEATH-RATE FROM DIPHTHERIA.

In reply to the question asked by Mr. Chancellor (published in The Hospital last week), Mr. John Burns has replied that in some of the last fifteen years there was an abnormal amount of diphtheria, and thus the deaths from this cause were higher than in some of the previous years. This, however, does not justify any inference that anti-toxin treatment is of little value. On the contrary, the treatment of diphtheria by anti-toxin has saved a large number of lives. With regard to that portion of the question referring to the tables published by the Asylums Board, Mr. Burns said it was true that the figures showed a larger decline in the case mortality of patients not treated with anti-toxin than of that of patients so treated, but no comparison can properly be drawn between the case mortality of these two classes of patients. The former class consists largely of persons suffering from a mild form of diphtheria, and, indeed, the few deaths that occur in this class are almost entirely those of patients who were too ill or who came into hospital at too late a stage in their illness to derive any benefit from the injection of anti-toxin.

The Editor, The Hospital.

---

The proprietors of Jeyes' Fluid have had the honour to receive the only Warrant of Appointment to his Majesty King George V. for disinfectants. Messrs. Jeyes have received no less than 135 gold medals and other awards, and have also held the royal appointments to her Majesty Queen Victoria and his Majesty King Edward VII.

Whites in the Tropics.—Whether white people can live—and work—in the tropics, writes our Australian correspondent, is a matter of extraordinary interest to Australia owing to the amount of country that lies in those regions and the people's determination to do without the labour of coloured races. Dr. Danes, lecturer in geography to the University of Prague, was commissioned some time ago to investigate the physical geography of limestone areas, especially those within the tropical zone. Dr. Danes journeyed over about 6,000 miles of Northern Queensland, and has now communicated his conclusions to Professor Edgeworth David, of the University of Sydney. Dr. Danes is strongly of opinion that the climate is healthy and the general geographical conditions favourable to the white races. But he thinks that two measures are necessary for the amelioration of present conditions and the prevention of immediate dangers : (1) Stringent medical sanitary supervision of aliens and the greatest possible restriction of their contact with whites or aboriginals, and (2) improvement of living conditions both of white and coloured population within the tropical and sub-tropical zones of Australia, especially with regard to housing, food, and water.

### Jottings.

Aided by brilliant weather, the Melbourne Hospital Saturday and Sunday collections on October 22 and 23 this year reached the sum of £8,119, with more still to come in. This is the highest sum realised for twenty-two years.

The Hon. Harry Lawson has promised to take the chair at the next festival dinner in aid of the funds of the Royal Hospital for Incurables, Putney Heath, and the Grocers' Company has kindly placed its hall at the disposal of Mr. Lawson for the dinner, which will take place on Thursday, May 4.

The new joint Infectious Diseases Hospital for the burghs of Dumfries and Maxwelltown, which has been built at Parkhead, Stoop, Dumfries, at a cost of about £6,000, has now been completed and opened. Altogether, including the two observation wards in the Administration block, there is accommodation for twenty-two beds.

The King has sent presents of game for the patients of St. Bartholomew's Hospital; St. Mary's Hospital, Paddington; Westminster Hospital; the Royal Hospital for Incurables, Putney Heath; the Middlesex Hospital; the Victoria Hospital for Children, Chelsea; St. Thomas's Hospital; the Charing Cross Hospital; the Brompton Hospital for Consumption; the West-End Hospital, Welbeck Street; the Kensington and Fulham General Hospital; and the Chelsea Hospital for Women.

The amount collected by the Hospital Saturday Committee on behalf of the funds of the Coventry and Warwickshire Hospital, Coventry, this year amounts to £3,300. This is £400 more than the amount collected last year, and is a record collection. The total handed over by the Committee of this Fund since its inauguration in the year 1874 amounts to £43,482 19s. 5d. The estimated population of the City is 100,000, it will therefore be seen that the donation just received works out at 8d. per head of the population.

Princess Alexander of Teck presided at a meeting of the Girls' and Boys' Guild of the Royal Waterloo Hospital last week, when it was announced that the proceeds of the *matinée* at His Majesty's Theatre on July 15 amounted to £732 17s., half of which has been handed to the Infants' Hospital, Vincent Square, and the other half to the Guild. The presentation of purses brought in £779, which will go towards the £1,000 required for the endowment of a cot. Her Serene Highness has allowed this cot to be dedicated to the memory of her infant son.

An extract from The Hospital with reference to a women's hospital as a sanctuary for mothers was read at the committee meeting of the Melbourne Women's Hospital on October 14. A copy of the extract was sent to the Chief Commissioner of Police, who of late has been "demanding" help from the women's hospital in tracing the mothers of deserted or murdered infants. As a fact, no "unmarried mother" is allowed to leave the Melbourne Women's Hospital without the committee satisfying itself that she has a home to go to or sending her to one.

Prince Alexander of Teck has consented to accept the proceeds from the sale of *Lusus*, a book of verse and prose, by Mr. Christopher Stone, on behalf of the Prince Francis of Teck Memorial Fund for the endowment of the Middlesex Hospital. Copies, price 5s. each (postage 4d. extra), may be obtained from Mrs. Christopher Stone, 177 St. James's Court, Buckingham Gate, from Mr. B. H. Blackwell, the publisher, 51 Broad Street, Oxford; or from Messrs. J. & E. Bumpus, Messrs. Truslove & Hanson, and Messrs. Hatchards, who have kindly undertaken to sell copies for the Fund.

The grandchildren of Charles Dickens and their friends will give an entertainment to the patients of the Royal Hospital for Incurables, Putney Heath, during January next.

The Barnato-Joel Memorial Buildings at the Middlesex Hospital are expected to be finished by next June, and it is understood that the Queen will open them about the middle of July.

His Majesty the King has sent twenty brace of pheasants to the Seamen's Hospital Society to be divided between the Dreadnought Hospital, Greenwich, and the Albert Dock Hospital.

The ball in aid of the Royal Waterloo Hospital, which had been arranged for Thursday last, December 1, in the Grafton Galleries, has been postponed on account of the General Election to Thursday, February 23.

Mr. D. D. S. Carter, hon. secretary of the Grantham Hospital, has received a cheque for £180, the result of the house-to-house collection in town and country. Of this sum Grantham contributed £40 odd, the country just over £90, and the hotel boxes nearly £10, a considerable advance on the total last year.

The King Edward Memorial for the United Provinces of India, which in population stand second only to Bengal, is to take the form of a sanatorium for tuberculosis, which, it is stated, will be started very shortly. Lieut.-Colonel C. C. Manifold, I.M.S., of Lucknow, Inspector-General of Civil Hospitals for the district, is in charge of the arrangements.

Prince Alexander of Teck announces that Mrs. D'Oyly Carte, Mr. J. Bannister Howard, and Mr. George Musgrove and several well-known amateurs will give performances of "The Belle of New York" in aid of the Prince Francis of Teck Memorial Fund at the Savoy Theatre from February 13 to February 18 next. Mrs. D'Oyly Carte has lent the theatre free of charge, and Miss Edna May (Mrs. Oscar Lewisohn) will appear in the name part. Applications for tickets should be made to the usual West-End agencies. Announcements of *matinées* of other plays in aid of the Memorial Fund will be made shortly.

Prince Christian and the Mayor of Windsor have issued a statement regarding the Windsor National Memorial, which is to take the form of a fund to pay off the debt upon King Edward VII. Hospital at Windsor, and to erect a statue to his late Majesty's memory in front of the hospital. The money for these two objects has been raised, the total amount having reached the splendid sum of £10,250, for which the Committee express their heartfelt thanks, and especially to Mr. A. W. Mayo Robson for his donation of £2,500. Hopes are now entertained that sufficient money may be raised to endow the hospital at least in part, and thus to ensure the carrying on of the work in the future. We hope that the many inhabitants in the neighbourhood who take a personal interest in the hospital will seize this opportunity of helping it thus practically. Subscriptions may be sent to his Royal Highness Prince Christian of Schleswig-Holstein, care of the Mayor, Guildhall, Windsor, and made payable to the Windsor Memorial Fund.

### TO HOSPITAL SECRETARIES AND OTHERS.

We invite contributions from any of our readers, and shall be glad to pay, according to length, for items of news for the institutional section (including appointments, resignations, gifts, etc.) which we may publish. All payments for manuscripts used are made as early as possible after the beginning of each quarter.

At a meeting of the Executive Committee of the Windsor National Memorial to King Edward held last Tuesday the Mayor (Councillor C. F. Dyson) reported that the subscriptions received amounted to £10,435, and the expenses to £195. The Mayor handed a cheque to the hospital's banker, who was present, for £6,914 to wipe off the debt on the King Edward VII. Hospital. Mr. Mayo Robson, the donor of £2,500 to the fund, was elected on the Committee.

### Gifts and Bequests.

Victoria Road (Baptist) Church heads the list of contributions to the Leicester Hospital Sunday Fund with £107.

Mr. Benjamin Levi, of Dalston, has bequeathed £1,000 to the Home and Hospital for Jewish Incurables, High Road, South Tottenham.

The treasurer of the Leicester Infirmary has received a legacy of £50 from the executors of the late Miss Mary Ann Roberts, of Redmile.

The Royal Dental Hospital, Leicester Square, has received £100 from Mr. R. McWhirter, as trustee of the estate of the late Mrs. Adelaide Roberts.

The Royal National Hospital for Consumption at Ventnor has received £1,000 from Mrs. Bell to endow a bed in perpetuity in memory of her late husband, Dr. J. Hougham Bell, a member of the board of the hospital.

We are very glad to state that the Secretary of the Queen's Hospital for Children was able to announce contributions amounting to £2,354 as received on the night last week when Lord Shaftesbury, president of this hospital, took the chair at the Hotel Cecil at a dinner in support of the institution's work in East London.

Mr. Thomas William Sorby, of Graham Road, Ranmoor, Sheffield, iron merchant and steel manufacturer, who left estate in the United Kingdom valued at £178,400 gross, has left a sum not exceeding £750 to endow a cot to bear his name in the Children's Hospital, Sheffield, and £100 each to the Sheffield Royal Infirmary and the Royal Hospital, Sheffield.

In response to an appeal issued by Major-General Lee for a fund to raise a memorial to the late King in connection with the Cardiff Infirmary, Lord Bute has agreed to subscribe 5,000 guineas. His grandfather was one of the principal founders of the infirmary, while his father gave the site on which the present buildings stand, and was also a large contributor.

Prince Alexander of Teck has received the following additional contributions to the Prince Francis of Teck Memorial Fund for the endowment of the Middlesex Hospital: Rear-Admiral David Beatty, D.S.O., £30; Lord Shaftesbury, £25; Lady Shaftesbury, £10; Mrs. Ferquharson, £10; Lord Burghclere, £10 10s.; the Chief Rabbi, £5 5s.; Colonel G. A. Percy, £50; Messrs. Joseph and John Vickers and Messrs. Hallows and Carter, £5 5s. each; Sir Rennell and Lady Rodd, £5; Sir Hugh Barnes, £5; Mr. Anthony Drexel, £100; "Per A. F. and O. S. F.," £100; Mrs. E. G. Watson, £20; Mrs. M. G. Wilson and daughter, £15; and the Hon. Sir Eric and Lady Barrington, £10.

Mr. Henry Andrade Harben, of Newland Park, Chalfont St. Giles, Chairman of the Prudential Assurance Company, Ltd., formerly Mayor of Paddington and member for South Paddington on the London County Council, who left estate valued at £342,740 gross and £306,364 net, has bequeathed £1,000 to endow a bed at St. Mary's Hospital, Paddington, £1,000 for the Medical School of that hospital, and £500 to Queen Charlotte's Lying-in Hospital. To the London County Council he left all his books, papers, prints, drawings, maps, and plans relating to the history or topography of London and the County of Middlesex, expressing the wish that these articles should be available for public study. This bequest is particularly opportune, since there is now a proposal to establish a London Museum as London's Memorial to King Edward.

The Executive Committee of the Clutton Memorial Fund issued an appeal early in the year to all old students of St. Thomas's Hospital and to a few friends of the late senior surgeon, Mr. H. H. Clutton. Contributions amounting to £692 13s. 6d. have been received during the past month. In view of the cordial response already made, the Committee feels that the appeal should now be sent to everyone connected with St. Thomas's Hospital, as it believes that many will welcome the opportunity of contributing to so good an object as this fund, which is to serve as a memorial to their old friend Mr. Clutton, and to form a nucleus of an endowment fund of the medical school in which he took so keen an interest. Cheques should be made payable to G. Q. Roberts, Clutton Memorial Fund.

---

To the incomplete list of medical candidates for Parliamentary honours, which we gave last week, the notable addition has to be made of Sir Victor Horsley, F.R.S., who is to contest London University in the Radical interest against the sitting Conservative, Sir Philip Magnus. Sir Victor's experience of successful electioneering in connection with the direct representation of the profession on the General Medical Council, as well as his intimate association with London University affairs and his robust personality, should make him a very dangerous opponent. That the advanced wing of his Party in Parliament would gain an active and strenuous recruit should he be returned is fairly certain; but there will be less unanimity in the profession whether as one of its representatives this well-known surgeon would be likely to reflect accurately the sentiments of the bulk of his fellow-professionals.

